# Assessing climate change vulnerability in a broadly ranging lizard: eco-physiological and thermal niche approaches

**DOI:** 10.1093/conphys/coag084

**Published:** 2026-11-02

**Authors:** J Erick Gómez-Campos, Rafael A Lara-Resendiz, Amando Bautista, Miguel A Domínguez-Godoy, Aníbal H Díaz de la Vega-Pérez

**Affiliations:** Doctorado en Ciencias Biológicas, Centro Tlaxcala de Biología de la Conducta, Universidad Autónoma de Tlaxcala, Carretera Tlaxcala-Puebla km 1.5 C.P., Tlaxcala 90062, México; Laboratorio Nacional de Biología del Cambio Climático, SECIHTI, Ciudad de México, México; Departamento de Ciencias del Agua y Medio Ambiente, Instituto Tecnológico de Sonora, Cd. Obregón, Sonora 85000, México; Centro Tlaxcala de Biología de la Conducta, Universidad Autónoma de Tlaxcala, Carretera Tlaxcala-Puebla km 1.5 C.P., Tlaxcala 90062, México; Laboratorio de herpetología, Departamento de Zoología, Instituto de Biología, Universidad Nacional Autónoma de México, A. P. 70515, C. P., Coyoacán 04510, México; Departamento de Sistemas y Procesos Naturales, Escuela Nacional de Estudios Superiores Unidad Mérida, Universidad Nacional Autónoma de México, kilómetro 4.5 Carretera Mérida-Tétiz, Ucú, Yucatán 97357, México

**Keywords:** Extinction, mechanistic models, protected areas, restriction hours, *Sceloporus grammicus*, thermal refuges

## Abstract

One of the principal causes of biodiversity loss and the sixth mass extinction is global climate change. Lizards, from tropical lowlands to cold high-mountain sites, are vulnerable to extinction due to their temperature dependence and the increasing environmental temperature. We modelled the projected local extirpation risk of the Mesquite lizard *Sceloporus grammicus* Wiegmann, 1828, the most widespread lizard species in Mexico. We integrated operative and microclimatic temperatures, eco-physiological traits (locomotor performance, and voluntary thermal range), distribution records and climate projections up to 2050 and 2070 under two climate change scenarios (Representative Concentration Pathways: RCP 4.5 and 8.5). Our eco-physiological model predicted a high risk of the projected local extirpation and thermally suitable area loss in both RCP scenarios and for both projections. We found a probability of projected local extirpation risk ranging from 25.1% to 39.4% and a probability of thermally suitable area loss ranging from 31% to 61% between the RCP 4.5 scenario in 2050 and RCP 8.5 scenario in 2070. Among the 90 terrestrial Protected Areas (PAs) evaluated, *S. grammicus* has historical occurrence in only 29 PAs for the current scenario (thermally suitable area of 51 250 km^2^) and only 23 PAs under RCP 8.5 for 2070 (29 505 km^2^) representing a reduction in thermally suitable area of more than 40% with only 23 historical occurrences within the Mexican PAs. Our results align with prior studies on this species and *Sceloporus*, showing that *S. grammicus*, despite a broad latitudinal and altitudinal distribution, is highly susceptible to the negative impacts of climate change. Mechanistic models are a powerful tool for evaluating reptile vulnerability, emphasizing physiological data as key to improving climate-response projections. These findings are directly applicable to conservation proposals.

## Introduction

Global warming is a major driver of biodiversity loss worldwide (Urban, 2015; IPBES, 2019; Reynolds *et al.,* 2019; Filippelli *et al.,* 2020; Domenici and Seebacher, 2020; Seebacher *et al.,* 2023) and approximately a quarter of all species worldwide face the risk of extinction (Parmesan, 2006; Sinervo *et al.,* 2010; Leung *et al.,* 2020; IUCN, 2023). Meta-analyses indicate that population declines, range reductions, as well as extinctions and changes in species distribution are accelerating, particularly among ectotherms such as amphibians and reptiles (Huey *et al.,* 2009; Diele-Viegas *et al.,* 2020). Assessing the impacts of global warming has become a conservation priority and various approaches have been developed to determine the vulnerability of biodiversity to climate change scenarios (Winter *et al.,* 2016; Enríquez-Urzelai *et al.,* 2019). Methodologies that incorporate multiple sources of information, such as physiological, ecological and distributional data using mechanistic and correlative approaches, have gained popularity due to their direct implications for conservation biology (Kearney *et al.,* 2010). Indeed, reviews have highlighted the importance of mechanistic models in relation to thermal ecology, particularly for the information and integration of data from physical models, thermal physiology, distribution and species-specific data (e.g. locomotor performance and voluntary thermal limits). These approaches provide mechanistically informed predictions of the responses of the species to and exposure to extreme temperatures under climate change (Buckley *et al.,* 2010; Kearney and Porter, 2020; Briscoe *et al.,* 2022; Stark *et al.,* 2023).

Recent studies have documented responses of reptile and amphibian species and populations to the increase in environmental temperature, habitat changes and thermal regimes particularly in desert regions of North America and Mexico (Biber *et al.,* 2023; Sinervo *et al.,* 2024). Reviews have shown the sensitivity of reptiles to climate change and their vulnerability (Böhm *et al.,* 2016). Consequently, mechanistic models have been widely used to evaluate species responses to climate change by the integration of physical models, physiological traits and climate variables (Buckley *et al.,* 2010; Sinervo *et al.,* 2024).

Mechanistic models integrate species-specific physiological traits, operative temperature (*Te*) and climate data to project and estimate how a thermal environment influences species distribution and vulnerability to climate change (Kearney and Porter, 2009; Buckley *et al.,* 2010). These approaches explicitly incorporate physiological mechanisms that determine activity, performance and thermal limitations, providing biologically grounded estimates of how species may respond to climate change. For example, Sinervo *et al.* (2010) developed an eco-physiological model that incorporates thermal niche approaches (i.e. the range of temperatures where the species is able to live, Bozinovic *et al.,* 2014; Gvoždík, 2018), where they demonstrated that several populations of *Sceloporus* lizards have become locally extinct in Mexico. Subsequent studies reported similar vulnerability patterns in other reptile populations under climate change (Pontes-da-Silva *et al.,* 2018; Caetano *et al.,* 2019; Lara-Resendiz *et al.,* 2021).

Lizard species with high thermal tolerances would be favoured by high environmental conditions and maintain activity under high temperatures (*Tropidurus torquatus*, Caetano *et al.,* 2019). Conversely, under climate change, species with relatively lower thermal tolerances may reduce activity and experience contractions in their geographic distribution. Opposing responses to warming associated with differences in thermal tolerance have been documented for *Phymaturus spurcus*, *Phyllotheca etheridgei*, *Phymaturus sinervoi*, *Phymaturus manuelae*, *Phymaturus tenebrosus*, *Phymaturus ceii*, *Phymaturus punae*, *Liolaemus montanezi, Liolaemus eleodori* and *Ctenoblepharys adspersa* (Ibargüengoytía *et al.,* 2021). In general, the vulnerability of a lizard species to climate change is determined by their thermal sensitivity, environmental exposure and life history traits, such as reproductive mode (Huey *et al.,* 2009).

The creation of protected areas (PAs) is one of the most crucial conservation strategies in the world (Santos-Barrera *et al.,* 2004; Greve *et al.,* 2011; Ochoa-Ochoa *et al.,* 2012). In general, the main goal of PAs is to preserve and protect biodiversity and unaltered ecosystems. By keeping these ecosystems intact within PAs, microclimatic conditions (humidity, precipitation and temperature) that are important for many species can be preserved. These features can help mitigate the impacts of anthropogenic activities and climate change. PAs maintain a wealth of flora and fauna, protect against extreme weather events, contribute to carbon sequestration and maintain ecosystem services (Díaz *et al.,* 2006; Greve *et al.,* 2011; CONANP, 2014; Álvarez-Yépiz, 2020). Therefore, conserving PAs is important for preserving many species (Lazcano *et al.,* 2012; Valdez-Rentería *et al.,* 2018; Cruz-Elizalde *et al.,* 2023). However, in Mexico, PAs are being affected by climate change and human activities, including illegal logging, fires, cattle grazing and agriculture, with a resultant loss of biodiversity (Sahagún-Sánchez *et al.,* 2013; Cruz-Elizalde *et al.,* 2015; Esperon-Rodriguez *et al.,* 2019). Such activities impact the long-term effectiveness of biodiversity conservation (Biggs *et al.,* 2008; Mansourian *et al.,* 2009). Some national-level assessments also indicate that many PAs may inadequately conserve biodiversity under current and projected climate change. For example, Ochoa-Ochoa *et al.* (2012) found that amphibian diversity in Mexico will decline due to climate change, particularly within PAs, diminishing their effectiveness. In addition, CONABIO (2020) notes that several federally protected regions are experiencing intensified environmental degradation from human activities, and that climate change diminishes the efficacy of the PAs.

Given these challenges, evaluating how species within PAs are affected under current and future climate change is essential. Several lizards have already been identified as vulnerable to climate change (Huey *et al.,* 2009; Sinervo *et al.,* 2010). In this context, *Sceloporus grammicus*, one of the most widespread lizards in Mexico, and a frequent inhabitant of PAs in Mexico, represents an ideal model to assess the effects of climate change on extinction risk using an eco-physiological and thermal niche approach (Díaz de la Vega-Pérez *et al.,* 2019; Chiu-Valderrama *et al.,* 2022).

In this study, we built species distribution models with three eco-physiological traits (i.e. field operative temperatures, locomotor performance and voluntary thermal range [VTR]). Using these models, we calculated (1) projected local extirpation risk under current (1950–2000) and future (2050, 2070) climate projections for two climate change scenarios (Representative Concentration Pathways: RCP 4.5 and 8.5), (2) periods of hours of activity and hours of restriction, (3) the thermally suitable area identified by the eco-physiological model in the current (1950–2000), and future (2050, 2070) climate projections for two climate change scenarios (RCP 4.5 and 8.5) and (4) the proportion of the thermally suitable area identified by the eco-physiological model within PAs for the current and future (2050, 2070) projections for two climate change scenarios (RCP 4.5 and 8.5).

## Materials and methods

### Study species

The Mesquite lizard, *S. grammicus* is a small species, with females and males weighing 5.3 ± 1.8 and 7.8 ± 2.6 g (mean ± SD), with a snout-vent length of 57 ± 7.2 and 64 ± 8.4 mm, respectively (Díaz de la Vega-Pérez *et al.,* 2019). It is the lizard species with the widest distribution in Mexico, ranging from the Lower Rio Grande Valley and Kingsville in southeastern Texas, USA, to southern Oaxaca (San Sebastián Coatlán), Mexico (Hall, 1973; Sites *et al.,* 1988; Sites *et al.,* 1992; Lemos-Espinal and Ballinger, 1995). It is a viviparous species that is highly adaptable to different environments, including urban areas, and is an excellent thermoregulator, which enables it to survive in thermally extreme habitats (Díaz de la Vega-Pérez *et al.,* 2019). *S. grammicus* is found in deserts, scrubs, pine, oak and high mountain moors, ranging from 1500 to 4440 m above sea level (m a.s.l.) (Hall, 1973; Sites *et al.,* 1988; Sites *et al.,* 1992; Lemos-Espinal and Smith, 2007). This species exhibits different local adaptations in morphology, thermoregulation, metabolic rates, microbiota, behaviour, natural history and reproduction, which allow it to inhabit contrasting environments mainly associated with elevation gradients (Montoya-Ciriaco *et al.,* 2020; Plasman *et al.,* 2020; Domínguez-Godoy *et al.,* 2020a, 2020b; González-Morales *et al.,* 2021, 2023; Chiu-Valderrama *et al.,* 2022).

### Study sites

The study was conducted on the volcano La Malinche at the Trans-Mexican Volcanic Belt. We selected three study sites on the eastern slope of the volcano in Tlaxcala, Mexico to represent the climatic variation of inhabited sites. The lowest study site was at 2600 m a.s.l. (19°14′N, 97°55′W; datum WGS 84). A cold climate prevails in this place with temperatures ranging from 14°C to 16°C (García, 2004). At this site, the lizards inhabit the rock walls of an abandoned construction surrounded by corn fields. The middle study site was at 3100 m a.s.l. (19°14′N, 97°58′W; datum WGS 84). A cold climate prevails in this place with temperatures ranging from 5°C to 12°C (García, 2004). It is an alpine grassland surrounded by *Abies religiosa* (Kunth) Schltdl. and Cham. and *Pinus montezumae* (Lamb.) forest. At this site, the lizards occupy fallen trees, boulders and rock walls. The highest study site was a rocky alpine grassland above the tree line near the top of the volcano at 4150 m a.s.l. (19°14′N, 98°02′W; datum WGS 84). A cold climate prevails in this place, with temperatures ranging from 2°C to 5°C. Here, the lizards inhabit natural volcanic rocks and *Juniperus monticola* (Martínez).

### Field methods

We replicated four physical operative-temperature models (*Te* models) to determine the field operative temperatures (*Te*) at each study site. According to Hertz *et al.* (1993), *Te* is defined as the range of potential body temperatures available to lizards in the absence of thermoregulatory behaviour (Porter and Gates, 1969; Baken, 1992). To determine the microclimatic characteristics, we placed *Te* models similar in shape and size to *S. grammicus* in typical perch sites (i.e. rocks and stone walls, Díaz de la Vega-Pérez *et al.,* 2019) under different sunlight conditions. We used polyvinylchloride (PVC) tubes (7 cm long, 1.5 cm wide) painted with 33% reflective grey spray paint as *Te* models of *S. grammicus*. These *Te* models (PVC tubes equipped with HOBO sensors) were previously calibrated in the field by measuring simultaneous body temperature of live *S. grammicus* and models across sunny and shaded microhabitats. The PVC models accurately mimicked the thermal characteristics of the lizards (*r* = 0.82, *P* < 0.001 see details in Díaz de la Vega-Pérez *et al.,* 2019; Domínguez-Godoy *et al.,* 2020a)*.* At each study site, we placed one data logger (HOBO® Pro v2-U23-003), equipped with two sensor channels inserted into the midsection of the *Te* models. The data logger recorded the *Te* of the lizard models at 30-minute intervals from 1 January 2017 to 23 October 2020 (1392 days) at the three study sites (Supplementary Materials S1 and S2). We took the measurements of *Te* from 08:30–18:00 h, which is the activity period of the lizards, as determined by the first and last sightings of this species at the different sites (more details, see Díaz de la Vega-Pérez *et al.,* 2019). We visited the dataloggers to extract the information every two months.

### Air temperature (*Ta*)

To estimate the average air temperature (*Ta*) at each study site, we installed a different model of data logger (HOBO® Pro v2-U23-002) at 150 cm above the ground level. Data loggers were permanently placed in shade microsites protected from direct solar radiation and located at a typical lizards perch site (i.e. rock wall at low elevation, trees at mid elevation and volcanic rocks outcrops at high elevations). This height approximates the thermal environment lizards experience during activity (see details in Díaz de la Vega-Pérez *et al.,* 2019; Domínguez-Godoy *et al.,* 2020a).

### Historical records

We obtained historical records of *S. grammicus* from the Global Biodiversity Information Facility (GBIF, 2019. Global Biodiversity Information Facility. https://www.gbif.org) and our fieldwork database. The records date from several decades (1800–2017) and correspond specifically to preserved specimens in scientific collections. The GBIF database was cleaned, by excluding duplicate records, records lacking geographic coordinates and those with incomplete or incorrect locality information based on published literature (Sites and Dixon, 1981; Sites *et al.,* 1992; Supplementary Material S3). To reduce spatial clustering, we filtered records to retain only one occurrence per 1 km^2^ grid cell, using the Mapinguari v0.4.1 (see details in Caetano *et al.,* 2020).

### Locomotor performance curves

To assess the potential range shifts of *S. grammicus*, we used locomotor performance data from Gómez-Campos (2017). Sprint performance was evaluated for lizards from the same three study sites (2600, 3100 and 4150 m a.s.l.) across five temperature treatments (20°C, 25°C, 30°C, 35°C and 40°C) to determine optimal performance (see Supplementary Material S4). These thermal performance curves were used as inputs to the projections, along with physiological parameters (VTR) that influence the activity periods and potential distribution of *S. grammicus* under current and future climate scenarios.

### Eco-physiological species distribution modelling

To project the extinction risk of *S. grammicus*, we used the method of Sinervo *et al.* (2010), which relies on VTR, historical records and bioclimatic layers. We adapted this approach by incorporating field-obtained thermal requirements of *S. grammicus* (i.e. VTR and operative temperatures, Díaz de la Vega-Pérez *et al.,* 2019 and this study), as well as thermal performance curves (Gómez-Campos, 2017), microclimatic temperatures (Díaz de la Vega-Pérez *et al.,* 2019; this study), historical records (see section below) and bioclimatic layers (WorldClim, http://www.worldclim.org). Using Mapinguari v0.4.1 in R v4.0.5 (R Core Team, 2021), we determined the probability of presence for current (1950–2000) and future conditions, applying bioclimate layers for 2050 and 2070 under two climate scenarios (RCP 4.5 and 8.5). For more details on Mapinguari and its methodology, see Caetano *et al.* (2019, 2020).

First, we quantified effective thermally available activity hours (*Ha*) and heat restriction hours (*Hr*) as mechanistic indicators of thermal constraints. Effective activity hours were obtained through a two-step calculation, as described below. To distinguish the initial model output from the derived effective activity estimate, we refer to the initial output as Ha_raw and the derived effective activity estimate as *Ha_real*; hereafter, *Ha_real* is denoted as *Ha* throughout the manuscript. The VTR was defined by the lower (*VTmin*) and upper (*VTmax*) voluntary thermal limits derived from body temperatures recorded from active lizards in the field.

In the R implementation, the initial activity metric (*Ha_raw*) counted the hours per day during which operative temperature (*Te*) was equal to or greater than *VTmin* (*Te* ≥ *VTmin*), without imposing an upper thermal threshold. *Hr* counted the hours per day during which *Te* exceeded *VTmax*, representing thermal restriction due to excessive heat. Consequently, hours classified as *Hr* were also included in *Ha_raw*. These metrics were calculated using physiological thresholds derived from the three study sites (2600, 3100 and 4150 m a.s.l). Generalized logistic functions (Richards method) describing the relationships between *Ha_raw* and *Ta* and between *Hr* and *Ta* were fitted in R using the Mapinguari package to generate the corresponding eco-physiological raster layers, following established mechanistic approaches (Sinervo *et al.,* 2010; Pontes-da-Silva *et al.,* 2018; Caetano *et al.,* 2020) We used active body temperature data reported by Díaz de la Vega-Pérez *et al.* (2019) for the period 2015–18, supplemented with field data collected at the same study sites from 2018 to 2020.

We then calculated effective thermally available activity hours by subtracting *Hr* from *Ha_raw* on a cell-by-cell basis: *Ha_real* = *Ha_raw* − *Hr*. At the hourly classification stage, this subtraction retains hours during which *VTmin* ≤ *Te* ≤ *VTmax*. The raster calculation therefore estimates the daily activity window after accounting for both the lower and upper voluntary thermal limits. The Resulting effective-activity raster, hereafter denoted as *Ha*, was used in the collinearity assessment and as the activity-hours predictor in the hybrid species distribution model. *Ha* represents thermally available time for activity rather than observed activity duration.

Locomotor performance data were recorded across five temperature treatments (20°C, 25°C, 30°C, 35°C and 40°C) for the three study populations. Since performance curves did not differ significantly among populations (*χ*^2^ = 10.31, *P* = 0.2437 see details in Gómez-Campos, 2017), data were pooled to fit a single species-level thermal performance curve using the PerfGAMM function in the Mapinguari package. This species-level curve was subsequently used by the EcoPhysRaster function to estimate locomotor performance for every raster cell. Specifically, EcoPhysRaster function mapped hourly operative temperature (*Te*) values onto the fitted performance curve, assigning an expected locomotor performance value to each hourly temperature. These hourly predictions were then averaged to produce a continuous spatial raster representing mean locomotor performance across the study area. EcoPhysRaster generated spatial layers of *Ha*, *Hr* and locomotor performance. These candidate eco-physiological raster predictors were subsequently evaluated using VIF analysis before model calibration.

To prevent multicollinearity among candidate environmental and eco-physiological predictors (effective activity hours, heat hours of restriction, locomotor performance and bioclimatic variables), we performed a formal collinearity assessment using the colinearity.test function implemented in the Mapinguari package (Caetano *et al.,* 2019, 2020). Predictor selection was based on Variance Inflation Factor (VIF) analysis using a threshold of 10. Variables with VIF values > 10 were excluded prior to model training. The final predictor set consisted of effective activity hours (*Ha*), locomotor performance and precipitation, all of which satisfied the VIF criterion. These variables represent complementary aspects of the species’ thermal ecology: *Ha* estimates the thermal available activity window after accounting for both voluntary thermal limits, locomotor performance reflects functional capacity across thermal conditions, and precipitation captures broad-scale environmental variation relevant to habitat suitability. *Hr* was excluded from final predictor set but retained as a complementary descriptive indicator of thermal stress.

We obtained bioclimatic layers representing minimum monthly temperature (tn), maximum monthly temperature (tx) and precipitation (pr) from the bioclimate data layers for future periods (2050 and 2070) from WorldClim version 1 at a spatial resolution of 30 arc seconds (1 km) (Hijmans *et al.,* 2005). We used WorldClim version 1 to ensure methodological consistency with the already established eco-physiological and mechanistic modelling frameworks for validating *Ha* and *Hr* in reptiles (Sinervo *et al.,* 2010; Pontes-da-Silva *et al.,* 2018; Lara-Resendiz *et al.,* 2019, 2021; Caetano *et al.,* 2020; Chiu-Valderrama *et al.,* 2022). In addition, we utilized two RCPs (4.5 and 8.5) from the Max Planck Institute (MPI-ESM-LR), which reflect different greenhouse gas emissions scenarios. The accuracy of the MPI-ESM-LR model for projecting and predicting climate change has demonstrated high effectiveness and reliability in its projections (Anav *et al.,* 2013; Giorgetta *et al.,* 2013). It has been used in various reptile groups, allowing inferences to be made about the effects of global warming on these organisms (Anav *et al.,* 2013; Ruiz-Barradas *et al.,* 2013; Pontes-da-Silva *et al.,* 2018; Sinervo *et al.,* 2018; Altamirano-Benavides *et al.,* 2019; Lara-Resendiz *et al.,* 2021). We modelled the probability of presence and the thermally suitable area identified by the eco-physiological model of *S. grammicus* for the current (1950–2000) and projected to future conditions (2050 and 2070) using three algorithms: Generalized Linear Models (GLM), Random Forest (RF) and Multivariate Adaptive Regression Splines (MARS). These algorithms represent complementary parametric, semiparametric and machine-learning approaches, respectively, and are able to capture both linear and complex nonlinear relationships between environmental (eco-physiological) predictors and species occurrence. Their combined use in an ensemble framework reduces model-specific uncertainty and enhances the robustness of projections (Barbet-Massin *et al.,* 2012).

We implemented the models using the ‘sdm’ package in the R v. 4.0.5 (Naimi and Araújo, 2016; R Core Team, 2021) incorporating the retained eco-physiological raster predictors (*Ha* and locomotor performance), precipitation and historical occurrences of the species. Before model fitting, the 517-occurrence data were randomly divided into a 70% training dataset and independent 30% test dataset for final evaluation. The training dataset was used to calibrate three algorithms: GLM, RF and MARS. The independent test occurrences were kept separate from model fitting and cross-validation and were subsequently used for final model evaluation (Phillips and Elith, 2010; Biber *et al.,* 2023; Biancolini *et al.,* 2024; Colyn *et al.,* 2025). Within the sdmData structure, 1000 pseudo-absences were generated using the geographically random sampling method (gRandom) implemented in the sdm package with remove = TRUE, to exclude points coinciding with occurrence records. The 1000 pseudo-absences were generated once as a single background dataset and were not partitioned into separate training and test subsets or regenerated for individual cross-validation repetitions or modelling algorithms. We ran five replicates for each statistical model using five-fold cross-validation to account for stochastic variability associated with repeated partitioning of the training data into calibration and validation subsets, following recommendations for regression-based species distribution models (Engler *et al.,* 2004; Barbet-Massin *et al.,* 2012; Naimi and Araújo, 2016; Pontes-da-Silva *et al.,* 2018), this generated 75 models (3 algorithms × 5 folds × 5 replicates). Finally, a projection margin of two geographic degrees was considered from the most extreme records of the base with clean records.

To validate the SDM, we used two approaches: (1) the area under the receiver operating characteristic curve (AUC-ROC) criterion, retaining models with AUC-ROC values ≥0.78, and (2) the true skill statistic (TSS), which is considered a critical threshold value that validates a positive diagnosis (Swets, 1988; Allouche *et al.,* 2006; Liu *et al.,* 2015) with values ≥0.44 (Naimi *et al.,* 2014; Hao *et al.,* 2020; details of the SDM are provided in Supplementary Material S6). Algorithms were implemented using their default parameters within the sdm package to maintain reproducibility and consistency with the standardized Mapinguari workflow. We evaluated the 75 models generated from the three algorithms (GLM, RF and MARS), five cross-validation folds and five replicates. Model performance was assessed using AUC-ROC and TSS values obtained from the independent 30% test dataset (test.indep). Models were retained for the final ensemble when they met both criteria of AUC-ROC ≥ 0.78 and TSS ≥ 0.44. Based on these criteria, 26 models were retained, including 25 RF models and one MARS model, whereas none of the GLM models met both criteria. We used the mean performance values across the 75 evaluated models (mean AUC = 0.786, TSS = 0.442) as model-retention criteria, rounded to AUC = 0.78 and TSS = 0.44. For each model, the suitability threshold was estimated using the threshold-optimization procedure implemented in the sdm package (opt = 2), which maximizes the sum of sensitivity and specificity. The mean model-specific threshold was 0.2844 and was rounded to 0.28 for binary classification of the ensemble predictions for current and future scenarios.

### Analysis of thermally suitable area and local extinction

To derive binary maps of thermally suitable areas for *S. grammicus*, we converted the continuous ensemble prediction into binary classification using an optimized suitability threshold of 0.28. Grid cells with predicted suitability values ≥0.28 were classified as thermally suitable, whereas cells <0.28 were classified as unsuitable. The same threshold was consistently applied across current and future scenarios to ensure strict comparability (Allouche *et al.,* 2006; Liu *et al.,* 2015). We assessed the variation in thermal conditions between the current and future scenarios by calculating the percentage of potential change in thermally suitable area (% change = [(S1—S0)/S0] × 100), where S0 represents the thermally suitable area under the current climate scenario and S1 under future conditions (Lara-Resendiz *et al.,* 2019, 2021). To evaluate the impact of these changes, historical records were classified as possible local extinctions if observed geographic locations occurred outside the predicted thermally suitable area for a given scenario. We then compared the number of such historical records between current and future scenarios. All spatial analyses were performed in QGIS 3.1 (QGIS Development Team, 2019), with raster layers processed at a spatial resolution of 30 arc-seconds (~1 km) using the coordinate system (WGS 84, EPSG:4326). We calculated the potentially thermally suitable area persistence (suitable in current and future scenarios), gains (suitable only in future scenarios) and losses (suitable only in the current) by comparing binary maps. This approach was applied to all geographic distribution and to PA analyses.

### Analysis of PAs

Our analysis included 90 Mexican terrestrial PAs (see Supplementary Material). Of these, 29 contain historical records of *S. grammicus*. We obtained PAs from the Comisión Nacional de Áreas Naturales Protegidas (CONANP, http://sig.conanp.gob.mx), classified into six categories: Biosphere Reserves, National Parks, Natural Monuments, Natural Resource Protection Areas, Flora and Fauna Protection Areas and Sanctuaries. U.S. PAs were excluded. We kept PAs boundaries static and overlaid them with thermally suitable area maps, using the same geographic coordinate system (WGS84; EPSG:4326) for all layers to maintain consistency. We used the classification of biogeographic provinces proposed by Morrone (2019) (Fig. 1a).

**Figure 1:**
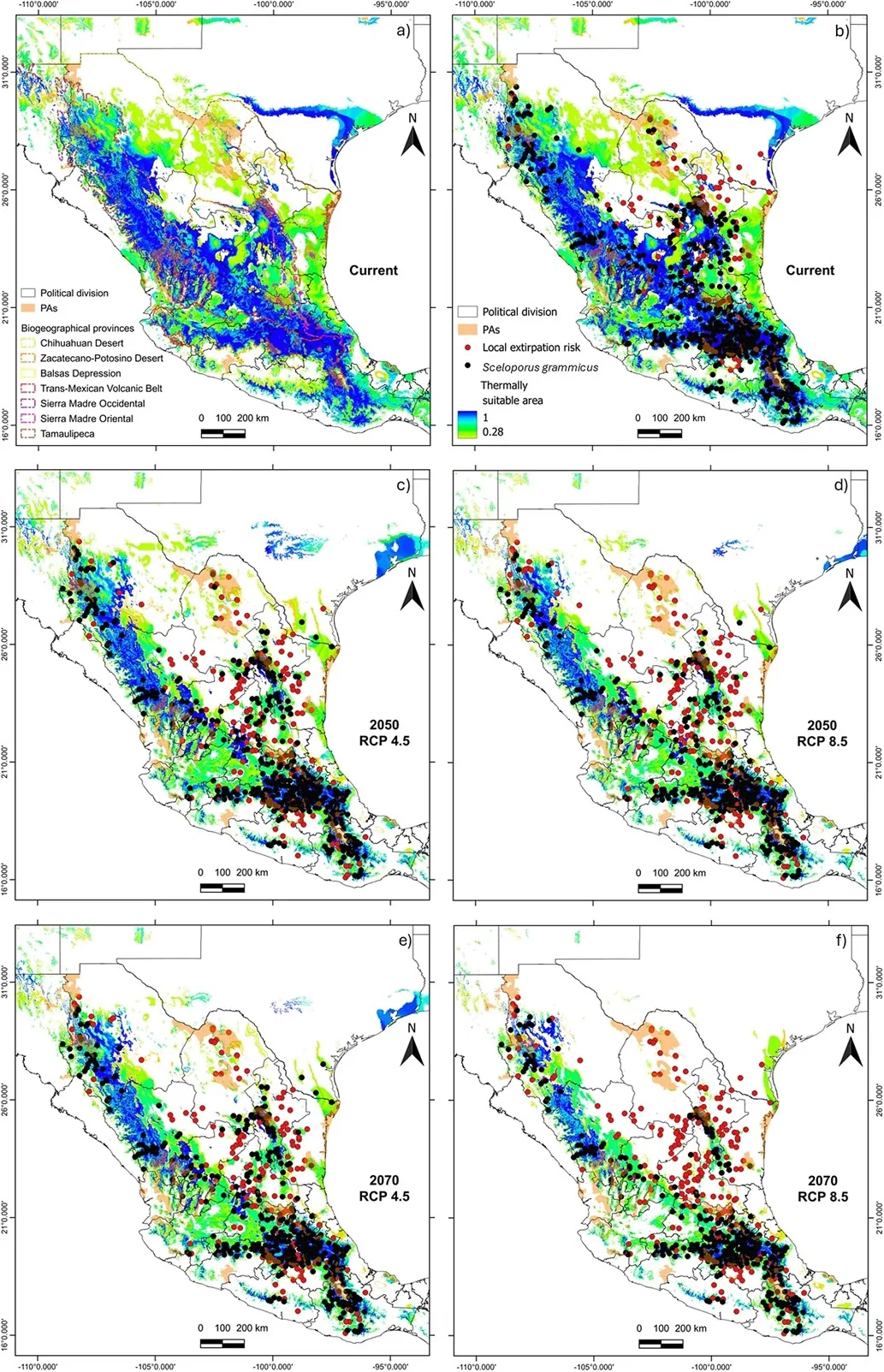
Spatial projections of continuous thermally suitable area and overlap with protected area boundaries under current and future climate scenarios (2050 and 2070, RCP 4.5 and 8.5).

## Results

### Species distribution model

We compiled a database with 18 648 total historical records of *S. grammicus*. However, after the cleaning process of 553 using the clean.points function, only 517 were retained, which included records from the USA to Oaxaca Mexico, with elevations ranging from 183 to 4225 m a.s.l. and latitudes from 16°05′N, 96°54′W to 30°22′N, 108°14′W. To reduce spatial sampling bias, it was verified that the geographic distribution between points was preserved in a historical record per cell minimum distance of 1 km resolution. This sample size was adequate to ensure calibration of the SDM across the entire thermally suitable area and distribution range of *S. grammicus*. In the future scenarios, the mean *Ha* and *Hr* range from 2.1 ± 0.9 to 2.5 ± 0.9 activity hours per day and from 4.3 ± 1 to 4.8 ± 1 restriction hour per day (Table 1; Supplementary Material S5).

**Table 1:** Thermally suitable area, local extinction, effective thermally available activity hours (*Ha*) and heat restriction hours (*Hr*) of *S. grammicus* in different scenarios

Scenario	Thermally suitable area (km^2^)	Thermally suitable area (%)	Local extinction (%)	*Ha*	*Hr*
Current	962 741		11.4	3.08 ± 0.9	3.6 ± 1.1	
2050rcp45	667 370	−31	25.1	2.5 ± 0.9	4.3 ± 1	
2050rcp85	528 910	−45	30.5	2.3 ± 0.4	4.5 ± 1	
2070rcp45	572 973	−40	27.6	2.4 ± 0.9	4.4 ± 1	
2070rcp85	377 702	−61	39.4	2.1 ± 0.9	4.8 ± 1	

The SDM based on the eco-physiological model yielded a total area of 962 741 km^2^ for the current scenario (Fig. 1b), representing the potential thermally suitable area for occurrence in the USA and Mexico, including Mexican PAs. Of the 75 models generated, only 26 met both model-performance criteria (AUC ≥ 0.78 and TSS ≥ 0.44) and were retained for the final ensemble. All current and future scenarios (2050 and 2070) indicate a substantial decrease in the thermally suitable area, with losses increasing with time. Under RCP 4.5 scenario for the year 2050, a projected loss of 31% (667 370 km^2^) was expected (Fig. 1c), increasing to 40% (572 973 km^2^) by 2070 compared to the current model (Fig. 1e). Under RCP 8.5, the projected loss is 45% (528 910 km^2^) (Fig. 1d) increasing to 61% (377 702 km^2^) by 2070 (Fig. 1f; Table 1).

The models used in the study indicate a high probability of local extinctions for *S. grammicus* for the current and future scenarios. Specifically, the current model showed a 11.4% of projected local extirpation risk (Fig. 1b). Under RCP 4.5, the probability increased to 25.1% in 2050 and 27.6% in 2070 (Fig. 1c and e). Similarly, under RCP 8.5, the probabilities of projected local extirpation risk were 30.5% in 2050 and 39.4% in 2070 (Fig. 1d and f; Table 1). The areas most affected by local extinctions include the Northern Highlands (Chihuahuan Desert), Southern Highlands (Zacatecano-Potosino Desert), Provincia Biogeográfica Tamaulipeca, Sierra Madre Occidental, Sierra Madre Oriental, Balsas Depression, Trans-Mexican Volcanic Belt and the southern Texas plain (Fig. 1a). The current scenario indicates the absence of thermally suitable areas under 183 m a.s.l. The models indicated that thermally suitable areas under both future scenarios are projected to occur only above 461 m a.s.l. Therefore, all distribution records below this threshold are outside the thermally suitable area and are considered local extinctions (Fig. S1).

Effective hours of activity (*Ha*) showed the greatest relative importance (0.50) in the final hybrid SDM, followed by locomotor performance (0.13) and precipitation (0.08) (Fig. S2A). The response curves showed a strong positive relationship between *Ha* and predicted suitability, with the steepest increase occurring between approximately 2.5 and 4 hours of effective activity (Fig. S2B). Suitability also increases gradually with precipitation, whereas locomotor performance showed a non-linear relationship with predicted suitability. These patterns indicate that areas providing longer effective activity periods were more suitable for the species.

### Protected areas

A total of 90 PAs were included in the analysis. Under the current scenario, 95% of these PAs currently maintain thermally suitable area for *S. grammicus*, covering 71 120 km^2^. However, historical records of the species are present in only 29 PAs (Fig. 1b). The RCP 4.5 scenario indicates a probable area loss of 24.3% (53 809 km^2^) in 2050 (Fig. 1c) and 31% (49 008 km^2^) in 2070 (Fig. 1e), compared to the current scenario. Similarly, under the RCP 8.5 scenario, projected area losses are 35% (46 180 km^2^) in 2050 (Fig. 1d) and 48.6% (36 514 km^2^) in 2070 (Fig. 1f), relative to the current scenario.

On the other hand, some PAs currently present thermally suitable areas but lack historical records of *S. grammicus*. Under the current scenario (Fig. 1b), a thermally suitable area of 19 870 km^2^ was found within 59 PAs. In contrast, under the RCP 4.5 scenario, projected gains in thermally suitable area are 11.3% (11 842 km^2^) in 2050 (Fig. 1c) and 11.7% (12 325 km^2^) in 2070 (Fig. 1e), occurring within 59 PAs in both years, relative to the current scenario. Similarly, under the RCP 8.5 scenario, projected gains are 10% (10 484 km^2^) in 2050 (Fig. 1d) and 6.6% (7009 km^2^) in 2070, with 57 PAs affected in 2070 (Fig. 1f; Table 2).

**Table 2:** Thermally suitable area in PAs with and without records of *S. grammicus* historical occurrence in different scenarios

Scenario	Thermally suitable area (km^2^)	Thermally suitable area (%)	Thermally suitable area with record (km^2^)	Thermally suitable area (%)	PAs with records	Thermally suitable area gain (km^2^)	PAs without records
Current	71 120		51 250		29	19 870	59
2050RCP45	53 809	−24.3	41 967	−18.1	25	11 842	59
2050RCP85	46 180	−35	35 696	−30.3	24	10 484	57
2070RCP45	49 008	−31	36 683	−28.4	24	12 325	59
2070RCP85	36 514	−48.6	29 505	−42.4	23	7009	57

In addition to these evaluations of the thermally suitable area identified by the eco-physiological model, we identified that potentially thermally suitable areas for *S. grammicus* were presented under the current and two future scenarios for both RCPs (4.5 and 8.5), although they showed a decreasing trend over time. Specifically, under the RCP 4.5 scenario, projected gains in thermally suitable area outside the current distribution are 16.2% in 2050 and 9.7% in 2070. Under RCP 8.5, the projected gain is 7.4% in 2050 and only 1.3% for 2070, representing the lowest gains among all scenarios (Fig. 2).

**Figure 2:**
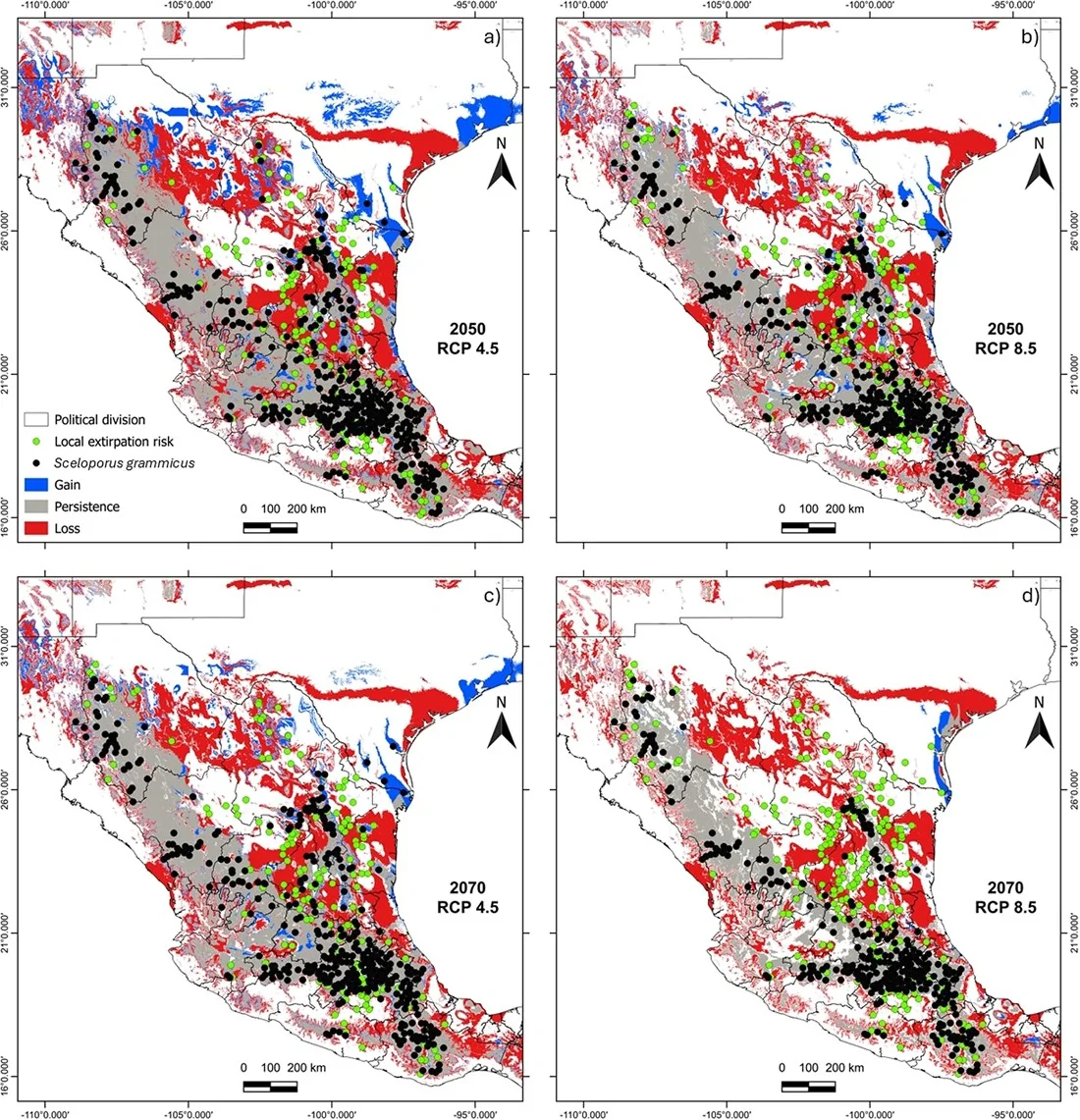
Projected changes in thermally suitable area for *Sceloporus grammicus* between current and future conditions (2050 and 2070, RCP 4.5 and 8.5) based on binary classification of ensemble suitability predictions. The figures show projected areas of gain (blue), persistence (grey) and loss (red).

## Discussion

Several studies have shown that rising temperatures due to climate change can reduce thermal suitable areas and accelerate extinction risk in reptiles, with the severity of the impact depending on physiological and ecological requirements (Huey *et al.,* 2010; Sinervo *et al.,* 2010; Vicenzi *et al.,* 2017; Biber *et al.,* 2023; Ameer *et al.,* 2025). In this study, we assessed the possible impact of climate change on the thermally suitable area identified by the eco-physiological model, including in PAs, and the geographic distribution of *S. grammicus* under current and future scenarios (2050 and 2070). Our eco-physiological models predicted a reduction in thermally suitable area from 31% under the RCP 4.5 scenario in 2050 to the highest loss of 61% under the RCP 8.5 scenario in 2070. The reduction in thermally suitable area suggests that *S. grammicus* could be approaching its physiological thermal limit under future scenarios. Similar reductions in thermally suitable area have been documented in other species of the genus *Sceloporus* with comparable thermal constraints (Huey *et al.,* 2010; Martínez-Méndez *et al.,* 2015; López-Vivanco, 2020; Muñoz-Nolasco *et al.,* 2023).

The sites showing a high probability of thermally suitable area loss and local extinctions in both RCP and future scenarios are located in the southern Texas plain, and primarily in northern Mexico, including several PAs with historical records, particularly across the Northern and Southern deserts, and mountainous ecosystems (Sierras and the Trans-Mexican Volcanic Belt). These areas are characterized by pronounced temperatures at low elevations and are arid and semi-arid zones in the desert regions of northern Mexico (García, 2004; Magaña *et al.,* 2012). In the case of the Trans-Mexican Volcanic Belt, below 2500 m a.s.l., semi-arid zones with temperate and semi-arid climates predominate, with the presence of *Quercus* trees and crops (García, 2004; Rzedowski, 2006). These findings are consistent with other lizard species that inhabit these ecosystems, where the ideal area is expected to decrease exponentially in the coming decades. A loss of 70–80% in habitat loss in *Crotaphytus antiquus* and *S. cyanostictus* reflects projected reduction in their geographic area by 75 and 84%, respectively, under a future scenario in 2050 (Gadsden *et al.,* 2012). In contrast, for *Phrynosoma cornutum*, 6% of its sites are projected to be locally extinct by 2050 and 18% by 2080. *P. modestum* is expected to lose 32% of its sites by 2050 and, alarmingly, 60% by 2080 (Gadsden *et al.,* 2012; Lara-Resendiz *et al.,* 2015).

Consistent with the patterns described above, *S. grammicus* appears to be highly vulnerable to the effects of climate change, as evidenced by the marked reduction in thermally suitable area and the increasing loss of climatically viable habitat across future scenarios. Similar conclusions were reported by Chiu-Valderrama *et al.* (2022), who also predicted a reduction in thermally suitable area under future climate scenarios. Their study projected the greatest decline (6%) under the RCP 8.5 scenario particularly in northern, central, and mountainous regions of Mexico. However, the reduction estimated (3.6–6%) in the study by Chiu-Valderrama *et al.* (2022) was substantially smaller than our findings (31–61%). These differences may be attributed to our methodological approach employed here, which explicitly incorporated thermal microenvironment data, thermal performance curves and active body temperatures from populations inhabiting contrasting thermal environments along a 1550-m elevational gradient. In addition, our analyses were based on an extensive sampling period of 46 months, allowing for a more detailed characterization of the species’ thermal ecology and its potential responses to future climate conditions. By contrast, Chiu-Valderrama *et al.* (2022) used a hybrid approach that integrated demographic models and correlative species distribution models.

This study was consistent with previous reports of viviparous lizards within the genus *Sceloporus*. For example, marked reduction in thermally suitable area and a high probability of extinction by 2070 were predicted for *S. serrifer* using both correlative and mechanistic models (Martínez-Méndez *et al.,* 2015). Likewise, a mechanistic modelling framework similar to that employed in our study indicated that *Sceloporus sugillatus* may experience a substantial reduction in thermally suitable area and a high risk of extinction, with local populations disappearing from some PAs by 2070, such as Corredor Biológico Chichinautzin, El Tepozteco and Parque Nacional Lagunas de Zempoala (Calderón-Chávez, 2021). The recurrent patterns of the loss of the thermally suitable area and high probabilities of projected local extirpation risk reported across these studies may be partly explained by the viviparous reproductive mode shared by these species. Viviparity has been proposed as an adaptation to cold environments, particularly at high elevations. However, prolonged exposure to elevated environmental temperatures or abrupt increases in body temperature in gravid females associated with climate warming may compromise embryonic development (Beuchat, 1986; Guillette Jr, 1993; Sinervo *et al.,* 2010). Consequently, increasing environmental temperatures may reduce the number of activity hours (*Ha*) while concurrently increasing the thermal restriction, thereby limiting opportunities for thermoregulation. Although *Hr* was excluded from final SDM calibration due to collinearity with *Ha*, its parallel increase provides a complementary mechanistic indicator of heightened thermal stress. The projected spatial contraction is primarily associated with the reduction in effective hours of activity (*Ha*) across warming scenarios, although locomotor performance and precipitation also contribute to the final SDM predictions. The combined thermal constraints may also reduce reproductive success, ultimately increasing vulnerability to climate change (Sinervo *et al.,* 2010). Warming may affect populations not only by reducing activity time but also by increasing the energetic costs of maintenance, growth and reproduction. Consistent with this, Wild *et al.* (2025) showed that warming can increase energetic demands while reducing foraging opportunities, using feeding demand as a mechanistic link between thermal exposure and energy budgets.

The projections also indicated that almost half of the thermally suitable area within PAs for *S. grammicus* will be lost (24.3–48.6% loss area between RCP 4.5 in 2050 and RCP 8.5 in 2070), indicating that even these PAs will be affected by climate change and may fail to fulfil their role as biodiversity reservoirs. Previous studies have also suggested that climate change could reduce optimal temperature conditions within PAs, leading to contractions in species distributions, which could reduce the availability of sites with thermally suitable area, resulting in ineffectiveness in these locations (Hanna *et al.,* 2005; Araujo *et al.,* 2011; Hao *et al.,* 2020).

To ensure the long-term persistence of thermally favourable sites for species such as *S. grammicus*, there is an urgent need to designate additional mountainous regions as PAs and improve the connectivity among them (particularly in areas of the Sierra Madre Occidental, Sierra Madre Oriental, Balsas Depression, Trans-Mexican Volcanic Belt). These regions exhibit extreme temperature ranges, including accelerated land-use change and fragmentation occurring in these areas (Arriaga *et al.,* 2000). However, the effectiveness of PAs is further compromised by other anthropogenic pressures including deforestation, land-use change, habitat fragmentation, fires, agriculture and livestock, which may drive extinctions even faster than climate change especially the areas of central Mexico (Romero-Báez *et al.,* 2024; Díaz-García *et al.,* 2025; Juárez-Ramírez *et al.,* 2025). At the national level, it has also been reported that within PAs there is a rapid deterioration of ecosystems due to constant human pressures (Keeley *et al.,* 2018; CONABIO, 2020; Hannah *et al.,* 2020; Stark and Galetti, 2024). Consequently, conservation actions are needed both inside and outside PAs in Mexico. Measures such as preventing land-use change, producing updated maps of land cover and vegetation, and maintaining current inventories of herpetofauna have proven highly effective for monitoring biodiversity and evaluating the conservation value of PAs (Figueroa and Sánchez-Cordero, 2008; Paredes-García *et al.,* 2011; Cruz-Elizalde *et al.,* 2023; Juárez-Ramírez *et al.,* 2025).

These findings support the idea that future climate change scenarios will provide new thermally suitable areas in terms of their thermal conditions. These thermally suitable new areas are located outside the current distribution of *S. grammicus*. These areas could represent potential refuges or opportunities for future colonization, and allow for potential expansion, as previously proposed, especially in higher elevation areas that are likely thermally suitable for *S. grammicus* and other reptile species (Nori *et al.,* 2015; Rodríguez-Muñoz *et al.,* 2016; Lara-Resendiz *et al.,* 2021, 2024). However, these new thermally suitable areas in terms of their thermal conditions may not be accessible for lizard colonization due to geographic barriers, limited locomotor ability or competition with resident species (Díaz de la Vega-Pérez *et al.,* 2022; Sinervo *et al.,* 2024). It is important therefore to monitor populations of *S. grammicus* in the USA and north of Mexico due to high extinction risk. Monitoring should use standardized mark-recapture programs, occupancy-based studies and range-change frameworks. These methods allow researchers to detect abundance trends, disappearances and potential colonization processes in relation to eco-physiological projections.

## Conclusions

Climate change exerts a significant negative impact on *S. grammicus*. It is likely to lead to a substantial reduction in thermally suitable areas and increase the likelihood of local extinction. It is unlikely that the distribution of this species in hot lowlands, deserts and mountainous regions of Mexico will be maintained. The potential extinction of the species in the USA is also a significant concern. Model projections indicate that under the most extreme future scenario (RCP 8.5) in 2070, *S. grammicus* will experience the greatest loss of thermally suitable area and the highest rates of local extinctions. Under such a scenario, there is a low probability of the lizards colonizing new sites, including certain PAs.

Although *S. grammicus* occupies a broad range of habitats across Mexico (occupying over 900 000 km^2^), including urban areas, our projections indicate up to 60% thermally suitable area loss under RCP 8.5 by 2070, highlighting pronounced vulnerability to climate change. Our findings emphasize the need for improved mitigation strategies and the establishment of new conservation areas to prevent further biodiversity loss. They also highlight the urgent need to integrate eco-physiological traits including thermal tolerances, operative temperatures and performance-related responses, into vulnerability assessments of Mexican reptile species, particularly for endemic *Sceloporus* lizard species with a restricted distribution and narrow thermal requirements. They underscore the importance of evaluating vulnerability under climate change scenarios, as the eco-physiological perspective may yield more accurate predictions for ectotherm species and inform more effective conservation actions and strategies.

## Supplementary Material

Web_Material_coag084

## Data Availability

Data are available in: https://osf.io/sndpw/overview.
