# Supplementary material for: Assessing climate change vulnerability in a broadly ranging lizard: eco-physiological and thermal niche approaches
Source: Conserv Physiol. 2026 Nov 2;14(1):coag084. doi: 10.1093/conphys/coag084 (PMC13729754; doi:10.1093/conphys/coag084)
Supplement: Supplementary file 1 [file web_material_coag084.zip › Suppementary_Figures.docx]

.

Fig. S1. Binary classification of predicted thermally suitable area under current and future climate scenarios (2050 and 2070, RCP 4.5 and 8.5; using a suitability threshold = 0.28).

Fig. S2. Relative variable importance (A) and response curves (B) of the predictors retained in the final ensemble species distribution model for *Sceloporus grammicus* (effective hours of activity (*Ha*), locomotor performance (total_perf), and total precipitation (total_prec).
